# Dengue hemorrhagic fever complicated with acute liver failure: a case report

**DOI:** 10.1186/s13256-017-1510-1

**Published:** 2017-12-08

**Authors:** Chamara Dalugama, Indika Bandara Gawarammana

**Affiliations:** Department of Medicine, University of Peradeniya, Peradeniya, Sri Lanka

**Keywords:** Dengue hemorrhagic fever, Liver, Hepatitis, *N*-acetyl cysteine, Packed cell transfusion

## Abstract

**Background:**

Dengue is a common arboviral infection with a clinically diverse spectrum of presentations. Although hepatic dysfunction is commonly identified in patients will dengue illness, acute liver failure is rare. The etiopathogenesis of hepatic dysfunction is multifactorial and related to direct viral invasion of hepatocytes, immunological factors and hypoxia particularly in cases of shock in dengue hemorrhagic fever. Ideal management of dengue-related hepatic dysfunction and acute liver failure is still debated.

**Case presentation:**

We report a 53-year-old Sri Lankan Sinhalese male with serologically confirmed dengue fever presenting with evidence of plasma leakage developing acute liver failure evidenced by deranged liver functions, coagulopathy and altered sensorium. In addition to the ‘standard care’, the patient was managed with intravenous *N*-acetyl cysteine and blood transfusions even in the absence of bleeding or dropping packed cell volume (PCV), targeting a higher PCV in anticipation of better oxygenation at tissue level. He made a full recovery with no sequential infections.

**Conclusion:**

*N*-acetyl cysteine and packed cell transfusion aiming at a higher PCV to maintain adequate tissue perfusion during shock may be beneficial in acute liver failure due to dengue virus. Large randomized trials should be carried out to establish the efficacy of these treatment strategies to support these observations and change the current practice.

## Background

Dengue fever is an arboviral disease that is endemic in Southeast Asia with four distinct serotypes [1]. Dengue illness has a diverse clinical presentation ranging from asymptomatic subclinical infection to severe multiorgan involvement and death [2]. Dengue illness can present with many unusual manifestations [3–5]. Hepatic dysfunction is a well-reported feature both in dengue fever and in dengue hemorrhagic fever (DHF). Liver involvement in dengue infection can be quite varied, ranging from mild to moderate elevation of serum transaminases to fulminant liver failure [6–13]. Various mechanisms are postulated to explain the hepatic dysfunction seen in dengue illness including direct viral damage, immunological injury and hypoxic injury due to reduced hepatic perfusion during shock [14–21]. Ideal management is still debated. We report an unusual case of an adult with dengue fever leading to acute liver failure and full recovery following treatment. The use of *N*-acetyl cysteine (NAC) [22–24], the use of serum lactate levels to monitor improvement and the use of packed cell transfusion to improve tissue-level oxygenation [25–27] are discussed as interesting management strategies.

## Case presentation

We report a 53-year-old Sri Lankan Sinhalese male from Kandy presented to Teaching Hospital, Peradeniya with a history of fever for 4 days. He was previously apparently well and a nonsmoker and a nonethanol consumer. He presented on the fourth day of an acute febrile illness with arthralgia, myalgia, retro-orbital pain and headache. On admission he was having postural dizziness, nausea, vomiting and right upper abdominal pain with severe anorexia. On examination he was oriented in time, place and person, but confused. He was not pale, but a mild tinge of icterus was noted in the sclera. The patient was dehydrated, but the capillary refilling time was less than 2 seconds with warm peripheries. His pulse rate was 96 beats/minute with a supine blood pressure of 120/80 mmHg. Standing blood pressure was 110/90 mmHg with narrowing of pulse pressure. The rest of the cardiovascular system was normal. Reduced breath sounds were noted in the right lung base but the rest of the lung fields were normal. He had marked tenderness over the right hypochondrium with a hepatomegaly of 4 cm below the costal margin. He did not have clinically detectable free fluid in the abdomen.

On admission to the medical unit the patient’s complete blood count showed hemoglobin of 12.6 g/dl, white cell count of 1.85 × 10^6^ cells/μl and platelet count of 36 × 10^3^ cells/μl. His transaminase levels were elevated: aspartate transaminase (AST) was 27,220 U/L and alanine transaminase (ALT) was 11,100 U/L. Serum bilirubin was 50 mg/dl with a direct fraction of 45%. The prothrombin time (PT) was 17.1 seconds (control 12 seconds) and activated partial thromboplastin time (APTT) was 142.7 seconds (control 29 seconds). Serum albumin was 30 g/dl and the corrected calcium value was 1.7 mmol/L (normal range 2.15–2.57 mmol/L). Venous blood gas showed a compensated metabolic acidosis with bicarbonate of 15 mmol/L and partial pressure of carbon dioxide of 20 mmHg. The venous lactate level was 5.8 mmol/L. Renal functions were within the normal range. Serum amylase was 145 U/L (normal range 1–137 U/L) and serum lipase levels were not available. Urine analysis showed trace protein, and three or four white cells with few granular casts. Bedside ultrasound scan revealed free fluid in perihepatic, subhepatic and pericholycystic spaces with marked gall bladder wall edema and a small right-sided pleural effusion. A diagnosis of DHF complicated with liver involvement was made.

The patient was moved to the high dependency unit and was catheterized. Fluid resuscitation was done initially using crystalloids. He was given a normal saline bolus of 7 ml/kg due to narrow pulse pressure and fluids were titrated according to blood pressure, urine output and packed cell volume (PCV). The patient was started on a third-generation cephalosporin and an intravenous proton pump inhibitor. He showed mild confusion and altered sleep cycle. In view of deranged liver functions, evidence of coagulopathy and altered sensorium, a possibility of acute liver failure with early encephalopathy was considered. Serum ammonia level measurement was not available at the hospital. The patient was started on oral metronidazole and lactulose. Intravenous vitamin K 5 mg was given. Blood sugar was checked every 4 hours and corrected. Serum calcium levels were monitored and corrected with intravenous calcium gluconate.

Dengue fever was confirmed by positive nonstructural protein 1 (NS1) antigen and the serotype was identified as DEN 2. Both dengue immunoglobulin M (IgM) and immunoglobulin G (IgG) were positive, suggesting a secondary infection with DENV. Because of severe liver involvement, alternative causes were sought. Tests for viral serologies including hepatitis A IgM, hepatitis B surface antigen and hepatitis C IgM were negative. Leptospira and rickettsial serology was negative and blood cultures were sterile. The patient denied taking a supra-therapeutic dose of paracetamol or any other hepatotoxic drugs. He had not taken any ayurvedic or herbal preparations prior to admission as an ailment for fever. Serology for Epstein–Barr virus (EBV) and cytomegalovirus (CMV) was not available in the hospital.

The patient’s PCV was maintained around 40% and he maintained more than 0.5 ml/kg urine output. Within the next 12 hours, further increase of transaminases was noted (AST 47,220 U/L, ALT 27,688 U/L) and the venous lactate level was raised to 7.8 mmol/L. The patient was started on an intravenous infusion of *N*-acetyl cysteine (NAC) at a rate of 100 mg/hour. The authors decided to transfuse packed cells to increase the PCV in order to improve the oxygen carrying capacity of the blood to relieve possible tissue-level hypoxia and to improve oxygenation at tissue level. The patient was given packed cells at a rate of 100 ml/minute to reach and maintain a PCV of 45% for 8 hours. Dramatic reduction of the venous lactate level was observed following packed cell transfusion. The patient’s postulated time window for plasma leakage (36–48 hours) was over by the following day. Gradual reduction in AST and ALT was noted. The PT and APPT were normalized. On day 5 of admission AST and ALT were less than 200 U/L, and NAC infusion was terminated and the patient discharged. He was in good health with normal liver enzymes, clotting profile and albumin at follow-up visits on day 7 and 2 weeks later.

Table 1 presents the trend of hematological, biochemical, clotting and blood gas parameters during the hospital stay and at follow-up visits.

**Table 1:** Trend of hematological, biochemical, clotting and blood gas parameters during the hospital stay and at follow-up visits of the patient

Parameter	Day 0	Day 1	Day 2	Day 3	Day 4	Day 5	Day 7	Day 14
Hb (g/dl)	12.6	13	14	16	14.4	14.1	13.3	13.5
WBC (10^6^ cells/μl)	1.85	2.06	2.66	4.37	8.25	12.4	10.6	8.9
Platelets (10^3^ cells/μl)	36	21	13	9	17	101	334	387
AST (U/L)	27,220	47,220	27,220	12,004	1732	108	88	40
ALT (U/L)	11,100	27,688	11,550	4590	1001	223	56	28
PT (seconds)	17.1		17.5		15.5			12
APTT (seconds)	142.7				56			25
Creatinine (μmol/L)	88				84			68
Albumin (g/dl)	30		31		35			42
Ionized calcium (mmol/L)	1.7		1.94		2.05			2.3
VBG bicarbonate	15	12	15.9	17	20			
VBG PCO_2_	20	18	24	26	28			
VBG lactate	5.8	7.8	4.8	2.5	2.2			
Amylase	145							

*Hb* hemoglobin, *WBC* white cell count, *AST* aspartate transaminase, *ALT* alanine transaminase, *PT* prothrombin time, *APTT* activated partial thromboplastin time, *VBG* venous blood gas, *PCO*_*2*_ partial pressure of carbon dioxide

## Discussion

Dengue is a common mosquito-borne viral disease among humans seen mainly in the Asia-Pacific region [1]. It can present with a diverse clinical spectrum ranging from asymptomatic infection or simple undifferentiated fever to DHF with multiorgan failure. Four distinct dengue viral serotypes (DEN 1–4) are known to cause illness. Infection with one serotype confers protection from reinfection with the same serotype, while reinfection with different serotypes confers no long-term protection and may even predispose the patient to plasma leak and worse clinical outcome [2]. No specific antiviral therapy is available for dengue fever. Close monitoring, detecting patients with plasma leak, meticulous titration of fluids to match the rate of fluid leak in the DHF group and managing complications of fluid leak are the main strategies for management.

Dengue infection can present with various unusual manifestations. Most of these manifestations of dengue fever are underreported, underrecognized or not casually linked to dengue fever, including hepatitis and liver failure [3], myositis [4], encephalitis and other neurological manifestations [5], and so forth.

Hepatic dysfunction is a well-recognized feature in both dengue fever and DHF. Liver involvement in dengue infection could be suspected in patients with dengue fever complaining of abdominal pain, nausea, vomiting and anorexia [6]. Hepatomegaly is present in both dengue fever and DHF but is more common in dengue fever [7]. Clinical jaundice has been detected in 1.7–17% of patients in various series [7, 8]. Mild to moderate increase in the transaminases is common in dengue fever and DHF. Souza *et al.* [9] described elevated transaminases in 74.2% of patients with serologically confirmed dengue illness (*N* = 1585) and 3.8% of them had transaminases at levels similar to acute hepatitis. Interestingly the greatest alterations were observed among females (*p* < 0.001), cases of DHF (*p* < 0.001) and cases with sequential infections (*p* = 0.001). Kuo et a. [10] and Nguyen *et al.* [11] observed that the level of aspartate aminotrasferase (AST) was higher than that of alanine aminotransferase (ALT). Kuo *et al.* [10] further observed that transaminases tend to decrease to normal levels within 3 weeks. AST released from damaged striated muscle, cardiac muscle and erythrocytes could explain the higher levels of AST than those of ALT in patients with dengue fever at an earlier stage [12, 13]. Therefore, a rise in AST might not be a true reflection of hepatic involvement.

The pathogenesis of liver injury in dengue infection is yet to be fully elucidated. Possible hypotheses include direct effects of the virus or host immune response on liver cells, circulatory compromise, metabolic acidosis and/or hypoxia caused by hypotension or localized vascular leakage inside the liver [14].

Studies have shown that dengue virus (DENV) readily infects liver cells in mouse models [15]. High levels of cytokines, particularly interleukin-22 (IL-22) and interleukin-17 (IL-17), were found in mouse models which may responsible for the cytokine-induced liver damage [16]. Sung et al. [17] observed that infiltration of hepatocytes by natural killer cells followed by T cells was associated with apoptosis of hepatocytes.

Histopathological studies of postmortem specimens of patients who had a fatal outcome have shown that the liver is congested with liver cell necrosis and apoptosis predominantly in midzonal and centrilobular areas, macrovascular steatosis and councilman bodies. Many postmortem reports show little or no inflammation [18, 19]. It is interesting that similar centrilobular necrosis is a typical finding in hypoxic hepatitis [20]. Considering the fact that a severe form of liver necrosis is seen among patients with DHF who present late with prolonged shock, we can postulate the fact that hypoxic injury due to reduced hepatic perfusion is probably an important contributor in causation of liver damage. On the contrary, a few cases of fulminant liver failure have been reported in the absence of shock [3]. Khongphatthanayothin *et al. *[21] reported an interesting case of liver failure from DENV infection with reversal of portal venous blood flow. They postulate that hepatic sinusoidal obstruction coupled with shock might be the underlying mechanism of liver failure in this disease.

In our case, the patient presented late during the leaking phase of dengue fever and on admission he had very high liver enzymes with deranged clotting and an elevated lactate level. He was resuscitated with fluids according to the PCV, urine output and hemodynamics. A liver failure regime with oral metronidazole and lactulose was started as he was in early hepatic encephalopathy. Acute liver failure in our case could be explained by initial direct viral damage and indirect immunological injury, and later contributed by splanchnic hypoperfusion due to plasma leak and compensatory redistribution of intravascular fluids and localized vascular leakage inside the liver. We believe the liver is more vulnerable than the kidney in this context because many mechanisms operate in liver injury as already mentioned. Hypoxia and hypoperfusion would have a greater impact on an already virally and immunologically damaged liver than on the kidney.

The patient was started on intravenous *N*-acetyl cysteine (NAC) infusion. NAC scavenges free radicals, improves antioxidant defense and acts as a vasodilator to improve oxygen delivery and consumption [22]. However, limited data are available in the literature regarding the efficacy of NAC in dengue fever-related liver dysfunction. A retrospective analysis of NAC in dengue-associated liver failure by Kumarasena *et al.* [23] showed that five patients who survived out of eight were in early (coma grade 1, 11) liver failure stage at the time when NAC was started. Habaragamuwa and Dissanayaka [24] reported another case of hepatitis following dengue successfully treated with NAC. Large randomized trials should be carried out to establish NAC efficacy along with appropriate dosage, timing and duration of treatment as there is no consensus on this. The authors decided to continue NAC in the patient until liver enzymes were near normal, anticipating the antioxidant and vasodilator effects might benefit the damaged liver.

During the critical phase of dengue fever the patient’s transaminase levels were increasing despite adequate fluid resuscitation with crystalloids. His PCV was around 40% at the time of admission and he had ultrasonic evidence of fluid leakage.

The authors considered that liver derangement could be partially due to the direct and indirect injury caused by DENV with a significant contribution from the reduced blood and oxygen supply to the liver although the blood pressure and PCV were static. This is possible as the liver is part of the gut and during early shock the hemodynamics are well maintained at the expense of compromised blood to the gut and liver. The circulatory shock, regardless of whether the etiology is cardiac or acute hypovolaemia or plasma leakage as in DHF, causes splanchnic hypoperfusion with no initial change in splanchnic oxygen consumption by diverting blood supply mediated by sympathetic adrenergic stimulation [25], both the liver and the gut are an efficient means of ensuring that vital organs are perfused during acute hypovolaemia [26, 27]. However, this could be counterproductive in a case of damaged liver by direct and indirect effects of DENV leading to further hypoxic damage due to reduced blood supply.

The authors hypothesized that increasing the PCV by transfusing packed cells may increase the oxygen carrying capacity of the blood and help the liver to recover. Based on this hypothesis, during the leaking phase of DHF the index patient was transfused 2 units of blood (1 unit approximates 400 ml of packed cells) at a rate of 100 ml/hour, targeting a PCV of 45%. Over the next 24 hours a reduction of liver enzymes was noted with marked clinical improvement. The venous blood lactated level dropped from a value of 7.8 mmol/L to 2.2 mmol/L at the end of 800 ml of blood transfusion over 8 hours. During the next 24 hours the patient’s PT and APPT normalized. His liver enzymes were less than 200 U/L on day 5 and NAC infusion was terminated. At the follow-up visits on day 7 and at 2 weeks the patient did not show any biochemical derangement of liver enzymes, and transaminase levels were in the normal range.

We considered the possibility of maintaining higher central filling pressures with crystalloids to maintain the splanchnic circulation, particularly in low-resource settings where blood products are not available. However, we believe this might worsen plasma leak into the pleural and peritoneal cavities and may be counterproductive. Blood, on the contrary, acts as a colloid, keeps the oncotic pressure within the vasculature and minimizes the plasma leak, and expands the vascular compartment and carries more oxygen.

## Conclusion

Although conclusions cannot be drawn from a single case report, we believe this case will bring light to treating physicians and researchers that increasing packed cell volume by transfusing blood will increase the oxygen carrying capacity and help a virally and immunologically damaged shocked liver to heal without heading toward fulminant liver necrosis and multiorgan failure followed by death. This hypothesis invites a large randomized trial in patients with severe hepatic dysfunction in dengue fever. One group treated with packed cell transfusion to keep the packed cell volume at a higher value compared with a group on ‘standard’ management where the packed cells are given only in a case of bleeding or dropping PCV with hemodynamic instability will add new knowledge and may change practice.
